# Biology of Beige Adipocyte and Possible Therapy for Type 2 Diabetes and Obesity

**DOI:** 10.1155/2016/9542061

**Published:** 2016-07-26

**Authors:** Fernando Lizcano, Diana Vargas

**Affiliations:** ^1^Center of Biomedical Research (CIBUS), Universidad de La Sabana, Chia, Colombia; ^2^Fundacion Cardioinfantil IC, Bogota, Colombia

## Abstract

All mammals own two main forms of fat. The classical white adipose tissue builds up energy in the form of triglycerides and is useful for preventing fatigue during periods of low caloric intake and the brown adipose tissue instead of inducing fat accumulation can produce energy as heat. Since adult humans possess significant amounts of active brown fat depots and their mass inversely correlates with adiposity, brown fat might play an important role in human obesity and energy homeostasis. New evidence suggests two types of thermogenic adipocytes with distinct developmental and anatomical features: classical brown adipocytes and beige adipocytes. Beige adipocyte has recently attracted special interest because of its ability to dissipate energy and the possible ability to differentiate itself from white adipocytes. Importantly, adult human brown adipocyte appears to be mainly composed of beige-like adipocytes, making this cell type an attractive therapeutic target for obesity and obesity-related diseases. Because many epigenetic changes can affect beige adipocyte differentiation, the knowledge of the circumstances that affect the development of beige adipocyte cells may be important for therapeutic strategies. In this review we discuss some recent observations arising from the great physiological capacity of these cells and their possible role as ways to treat obesity and diabetes mellitus type 2.

## 1. Introduction

Diabetes mellitus type 2 (DM2) is a chronic disease, the incidence of which has increased dramatically in recent years. The consequences of diabetes are devastating due to side effects that occur in the cardiovascular system [1, 2]. Both DM2 and obesity have become a pandemic that has begun to appear in developing countries and in developed countries the public measures to improve lifestyle habits have not reduced significantly the incidence of these diseases [3, 4]. While one approach to DM2 is based on improving life style including exercise and reduction of weight, therapeutic advances are multiple and include drugs protecting the function of the pancreatic beta cells, increasing insulin sensitivity, rising the excretion of sugar in the urine, and modulating satiety levels via the hypothalamus [5, 6]. The current therapeutic options for obesity are limited, given the undesirable side effects presented by many of the therapies employed to date. In addition, in many countries, only a few medications have been approved and the therapeutic effectiveness of these is not as good as expected [7]. A therapy that can reduce the accumulation of calories or increase energy expenditure to improve insulin sensitivity, reduce weight, and preserve the activity of the pancreatic beta cell is desirable but has not yet been discovered. Potential new players in the area of obesity treatment are under evaluation, and like the approach to diabetes these therapies involve drugs that enhance the balance of the satiety level in the hypothalamus and increase caloric expenditure [8–10].

Given that obesity and DM2 have precise events in common, such as energy balance, stimulation of thermogenesis, and calorie expenditure, a possible therapeutic strategy could be the manipulation of the adipose tissue that controls the balance between the accumulation of energy and the production of heat. Utilizing these parameters, from this perspective, the treatment of DM2 and obesity could be through the modulation of the physiology of the adipocyte precursors.

In recent years, it has been observed that the adipose tissue is more dynamic than previously believed [12]. The classical white adipose tissue (WAT) builds up energy in the form of triglycerides and is useful for preventing fatigue during periods of low caloric intake. The brown adipose tissue (BAT) is more energetically active, with a greater number of mitochondria and energy production in the form of heat, which controls homeostasis during periods of low temperature and hibernation [13]. In human adults, it is believed that white adipose tissue predominates because most of brown adipose tissue is seen only in the first few months of life [14]. However, it is obvious that an adipose tissue similar to brown adipose tissue can be seen in adults when they are subjected to low temperatures or sympathetic stimulation [15]. Despite the fact that the characteristics of this type of tissue are the same as brown adipose tissue, it is likely that these characteristics correspond to an adipose tissue variant, an energy asset that has been termed beige adipose [16–18].

While all adipocytes are mesenchymal in origin, an appreciable diversity arises during the process of differentiation; in fact BAT, in some way, has more in common with muscle cells of mesenchymal origin than with WAT [19–23]. In addition, the precursor cells of white adipose tissue can be modified with different factors to give rise to adipose tissue that is more energetically active [24, 25], Table 1 Multiple factors may modulate the process of differentiation of beige adipocytes from adipocyte precursor cells. Extracellular signals include the activation of the sympathetic nervous system, the cells of the immune system, and epigenetic variations [26] that influence the transcription of specific genes [27].

## 2. The Origin of Beige Adipocyte

The beige adipocyte is a type of adipose cell described by the ability to induce these cells to produce heat and increase energy expenditure. The beige adipocyte can be considered phenotypically as a fat cell that possesses characteristics between those of the white fat cell, an accumulator of energy, and the brown cell, which produces heat [28].

The origin of the beige adipocyte is complex; some beige adipocytes arise in epididymal white fat from precursors that express platelet-derived growth factor receptor alpha PDGFR*α*, CD34, and spinocerebellar ataxia type 1 (SCA1) proteins [29–34]. Beige adipocytes may be obtained from myogenic factor 5- (*Myf5*-) negative precursors of inguinal white fat tissue. However, a group of these adipocytes originated from* Myf5*-positive precursors have been reported [33]. Recently, it has been described that some beige adipocytes are Myosin Heavy Chain 11- (*Myh11*-) positive, which is a selective marker of smooth muscle cells [35]. All of these results indicate that beige adipocytes have a cellular origin different from the classical brown adipocyte.

Another feature of beige adipocytes is the capacity to have a flexible phenotype. While development of beige adipocytes is highly inducible from precursor cells, there is evidence that mature white fat cells can be changed to beige adipocytes through specific factors [36, 37]. Whether this phenomenon represents a real transdifferentiation, direct transformation of white adipocyte cells to beige adipocyte cells, or corresponds to a beige adipocyte that was probably previously hidden among the white fat cells is a matter of debate [38]. One of the issues involving any special assessment in human adults is whether the adipocytes in which thermogenic properties have been detected are in reality brown adipocytes or beige adipocytes [39, 40]. According to recent studies, most of these adipocytes in the adult are considered to possess the characteristics of beige adipocytes. However, in zones like the posterior part of the neck still there are adipocytes that conserve brown phenotype [20, 39, 41–43].

## 3. Inducers of Beige Adipocyte

### 3.1. Induction of Beige Adipocyte by Cold

The primary inducer of beige adipocytes is a reduction in temperature; this effect is obtained mainly through the stimulation of sympathetic nervous system. The general consensus is that cold stimulates a greater release of catecholamines by the nervous system, an event that stimulates thermogenesis through the activation of protein kinase A (PKA) and p38 mitogen-activated protein kinases (p-38 MAPK) pathways followed by the activation of uncoupling protein 1 (UCP1) and phosphorylation of the specific factors PPAR gamma coactivator 1 alpha (PGC-1*α*), cAMP response element-binding protein (CREB), and activating transcription factor 2 (ATF2) [14, 15, 44, 45].

**Figure 1:**
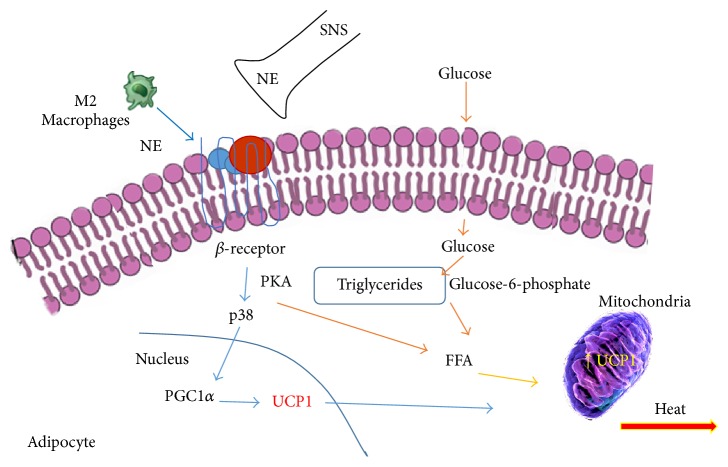
The effects of cold on the induction of thermogenic active adipocyte. The secretion of norepinephrine (NE) directly from nervous sympathetic system or indirectly through catecholamine secretion by macrophage type 2 can stimulate glucose uptake in fat cells and improve the regulation of carbohydrate. Both glucose and triglycerides are used by UCP1 protein to increase thermogenesis. FFA: free fatty acid; M2: macrophages 2, SNS: sympathetic nervous system; PKA: protein kinase A; UCP1: uncoupling protein 1; PGC-1*α*: peroxisome proliferator-activated receptor-gamma coactivator alpha 1.

Additionally, increase of norepinephrine after cold stimulation is associated with the immune system. In fact, a greater number of type 2 macrophages with capacity to produce catecholamines have been observed in the subcutaneous fatty tissue after exposure to the cold [18, 46], Figure 1.

The cold as an inducer of the beige adipocyte has two functions, an initial effect that the cold has on the precursors of the adipocytes and the role that the cold plays in the mature cell during the process of beiging. While the cold effect in part may be mediated by IL-4 and its receptor IL-4Ra in the adipocyte precursors, in mature adipose cells a reduction in the IL4Ra is observed and probably this effect may include diverse mediators, including IL-33 and Met-enkephalin [47, 48].

Rich environmental stimuli (physical and social stimulation) play important roles in the dynamics of beige adipocyte development. An environmental stimulus may produce an increase in the secretion of catecholamines via the hypothalamic secretion of brain-derived neurotrophic factor (BDNF) [49]. The level of the neuron growth factor inducible VGF is increased in rich environments, and VGF appears to act as a mediating factor in the BDNF pathway [50].

### 3.2. Influence of Physical Exercise

Several types of exercise can increase calorie expenditure through a process of beiging. The main mediator of this process seems to be PGC-1*α*, which influences myogenesis, mitochondria, and oxidative phosphorylation [51–53]. Some proteins produced in the muscle can influence the metabolism of the fat cell. The protein derived from fibronectin type III domain containing protein 5, IRISIN, and the protein denominated as meteorin-like protein precursor (METRNL) can lead to the process of beiging in different ways [54, 55]. While Irisin regulates the expression of specific genes in beige adipocytes [56], METRNL increases the activation of type 2 macrophages through eosinophils [57]. The proinflammatory interleukin IL-6 can also increase the process of beiging and induce an increase in calorie expenditure [58]. It is likely that a product of anaerobic exercise such as lactate can influence the process of beiging; this possibility requires future study [59].

### 3.3. Interleukins

Obesity has been considered to be a condition of slight chronic inflammation, and interleukins can influence the function of fat cells in diverse ways. Proinflammatory interleukins that are increased in obese people, such as TNF-*α*, IL-1*β*, and IL-6 secreted by type I macrophages, induce undesirable effects leading to cardiovascular complications in patients with obesity [60–63]. In contrast, in thin subjects and with specific stimuli, it is possible to generate a change in the population of macrophages by increasing the number of anti-inflammatory type II macrophages [64]. These macrophages may maintain sensitivity to insulin and remodeling of the extracellular matrix [65]. Type II macrophages are activated by T-lymphocytes and eosinophils [66, 67]. In mice, Th2 lymphocytes secrete the anti-inflammatory interleukin IL-10, which improves insulin sensitivity by blocking the action of TNF-*α*. In addition, the eosinophils that migrate to the adipose tissue can maintain the activity of the M2 macrophages via the secretion of IL-4 and IL-13 [68–70]. The presence of type 2 innate lymphoid cells (ILC2s), which act as T helper lymphocytes and produce IL-4, IL-5, and IL-13, has attracted particular interest because a reduction of these cells in adipose tissues is associated with obesity in mice and humans [71]. Recently it has been observed that IL-33 is required for the maintenance of ILC2s in the white fat cells and for the development of the beige adipose phenotype [72]. IL-33 can induce the production of IL-4 by eosinophils and increase the production of Met-enkephalin by ILC2s [47, 73].

### 3.4. Noncoding miRNA

MicroRNAs (miRNAs) are a class of short noncoding RNAs that alter the expression of genes. Despite the fact that the main effect of miRNAs is the inhibition of the translation machinery, an increase in activity has been observed in some cases. A large number of miRNAs have demonstrated the ability to regulate the differentiation of beige adipocytes from precursor cells. However, only one group has shown a specific effect with possible clinical relevance. Some microRNAs have the ability to negatively regulate the activity of PR domain containing 16 (PRDM16), including miR-133, miR-193b, and miR-365 [74–76]. miR-93 acts as a negative regulator of adipogenesis by influencing adipocyte precursors via the modulation of sirtuin 7 (Sirt7) [77].

Other miRNAs, such as miR-196a, can increase the production of beige adipocytes by blocking the expression of homeobox C8 (HoxC8), which negatively regulates the activity of CCAAT/enhancer-binding protein beta (C/EBP-*β*) [78]. The influence of miRNAs is seen in the repression of the activity of molecules such as phosphodiesterase 1*β* (PDE1*β*) and receptor interacting protein 140 (RIP140) by miR-378 and miR-30, which induces the development of beige and brown adipocytes [79, 80]. miR-34 may suppress the differentiation of beige adipocytes by reducing the activities of sirtuin 1 (Sirt1) and fibroblast growth factor 21 (FGF21) in mice [81]. Some experiments in human adipocytes have shown that miR-26 activates the differentiation of beige adipocytes [82].

Similarly long noncoding miRNAs, such as BATE1 and Blinc1, are required for the formation of beige and brown adipocytes through the production of related nucleoproteins that influence the activation of thermogenic genes [83].

### 3.5. Endocrine Factors and Metabolites

There are a significant number of endocrine factors that have the ability to regulate the occurrence of beige and brown adipocytes. Factors such as BMP7 [84, 85], BMP8b [86], FGF21 [87, 88], prostaglandins [89, 90], natriuretic peptides [91], and *β*-aminoisobutyric acid (BAIBA) [53] can influence the differentiation of beige adipocytes. All of these factors, which are capable of increasing caloric expenditure by various mechanisms, have protective effects regarding obesity in animals fed a high-caloric diet and improve glucose homeostasis and insulin sensitivity.

The activation of the nuclear receptor PPAR*γ* has a strong effect on the differentiation of adipose cells and is involved in the differentiation of all types of fat cells. PPAR*γ* agonists have been used clinically to improve insulin sensitivity; however, undesirable side effects have limited the therapeutic use of these compounds [92, 93]. Recently, selective agonists of PPAR*γ* have been shown to control the expression of genes through enzymatic modifications such as phosphorylation or deacetylation [94–96]. The mechanism by which some ligands of PPAR*γ* induce the transcription of genes involved in the process of differentiation of the beige adipocytes includes the activation of Sirt1, a histone deacetylase NAD^+^-dependent protein, which might be influenced by PPAR*γ* itself and combine with PRDM16 [97, 98]. It has been observed that an increase of Sirt1 in adipose tissue improves obesity by increasing thermogenesis.

Thyroid hormones induce an increase in the dedifferentiation of beige adipocytes through a direct regulation of UCP1 promoter [99]. Estrogen may affect the adipocyte cell in different ways. In brown adipocyte cell the estrogen receptor alpha stimulation (ER) can increase the expression of UCP1 by rising PGC-1*α* coactivator through AMPk, while in white adipocyte ER*α* activation by estrogen reduces lipoprotein lipase and increases beta-adrenergic receptor activity [100].

### 3.6. Epigenetics and Regulators of Chromatin

Covalent modifications on histones induce a change in the conformation of chromatin, which results in a change in the expression of genes. During the differentiation process of beige adipocytes, there are several factors that modify the chromatin and thus determine the function of genes that increase caloric expenditure and determine a beige adipocyte phenotype.

The histone lysine demethylases KDM4a/JMJD2a, -JMJD2c, and KDM3a/JMJD1a may interfere with the differentiation of adipocytes by different mechanisms. Jmjd2a and Jmjd2c can modify the expression of genes controlled by the nuclear receptor PPARg [101, 102]. KDM3a bides within the SWI/SNF complex in the chromatin and controls the activity of the *β*1-adrenergic receptor [103]. Mice with a* jhd1a *knockout have an obese phenotype due to the increased oxidation of fatty acids [104, 105].

The histone methyltransferase EHMT1 forms a complex with PRDM16 and is required for the determination of the production of the beige lineage [106]. EHMT1 increases the production of UCP1, and the deletion of this gene causes insulin resistance and obesity in mice, while haploinsufficiency is related to obesity and insulin resistance in humans [107, 108].

Histone deacetylase 1 (HDAC1) negatively regulates the thermogenic program of brown adipocytes [109]. Coordination between the inhibition of HDAC1 and the activation of the histone demethylase lysine-specific demethylases (KDM6b/JMJD3a) and KDM6a/UTX activates brown adipocyte genes and prevents the appearance of obesity [110, 111].

**Figure 2:**
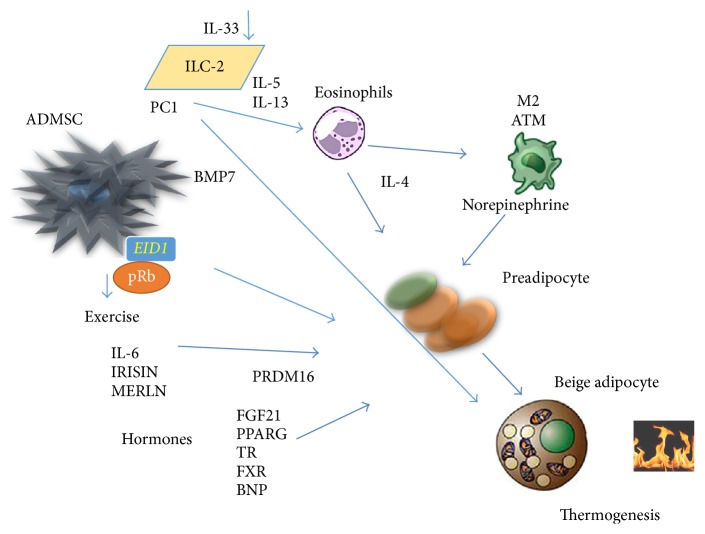
Determining factors in differentiation of beige adipose cell. The adipose mesenchymal cells can be influenced by the retinoblastoma protein (pRb) and take decision to differentiate into fat cells when pRb is blocked. The EID1 protein among others can determine the differentiation of beige cells adipocytes from mesenchymal stem cells (see [11]). BMP7 triggers production of mesenchymal adipose cells to brown adipose cells. Both exercise and some hormones can increase the capacity of adipose stem cells to differentiate into beige adipocytes. Recently, it has been observed that cells of the innate immune system type 2 can secrete interleukins stimulating the production of IL-4 by eosinophils and norepinephrine production through macrophage type 2. IL-33 has the ability to activate the differentiation of beige adipocytes directly. ADMSC: adipose mesenchymal stem cell; PC1: Prohormone Convertase 1; ILC-2: group 2 innate lymphoid cells; BMP7: bone morphogenic protein 7; EID1: EP300-interacting inhibitor of differentiation 1; M2 ATM: adipose tissue macrophage 2; FGF21: fibroblastic growth factor 21; PPARg: peroxisome proliferator-activated receptor gamma; TR: thyroid receptors; FXR: farnesoid X receptor; BNP: brain natriuretic factor.

The retinoblastoma protein (pRb) is a regulator of the differentiation of mesenchymal cells to different levels [112]. A deficiency in pRb increases the differentiation of the mesenchymal cell precursors into the brown adipocyte lineage with a reduction in the differentiation into osteoblasts or white adipocytes [113]. In addition, a blockage of pRb activity can control the decision of adipocyte precursors to progress toward the development of beige adipocytes. EP300-interacting inhibitor of differentiation 1 (EID1/CR1) can reduce the activity of pRb and induce differentiation to beige adipocytes [11, 114]. p107, a cell-cycle regulator that belongs to the pRb family, has demonstrated an important role in the decision of mesenchymal stem cells in the differentiation of beige adipocytes [115], Figure 2.

### 3.7. Potential Therapeutic Use in Humans

A characteristic of white adipocyte tissue (WAT) is the capacity to change its dimensions. When an increase of caloric intake or reduction of physical activity produces positive energy balance the adipocyte cell becomes hypertrophic [116, 117]. In obese and diabetic persons, the expansion through adipocyte hypertrophy is accompanied by a shift to an adverse adipokine secretory profile, which typically includes an elevated array of proinflammatory factors, such as TNF, IL-1*β*, IL-6, IL-8, resistin, and monocyte chemoattractant protein 1 (MCP1), with a parallel reduction in anti-inflammatory factors, such as IL-10, adiponectin, and FGF21 [118]. In obesity, WAT may become severely dysfunctional and thereby fail to appropriately expand to store surplus energy. At the whole-body level, this dysfunction results in ectopic fat deposition in other tissues that regulate the metabolic homeostasis such as hepatic, pancreatic, and skeletal muscle tissues. The low inflammatory condition in the obese person reduces the insulin sensitivity and causes the majority of cardiovascular complications [13, 119].

There have been many clinical studies in human adults that suggest the beneficial effect of activating beiging from the WAT. Yoneshiro et al. [120] showed that daily 2-hour cold exposure at 17°C for 6 weeks resulted in increases in BAT activity and cold induced increments of energy expenditure and a concomitant decrease in body fat mass. Chondronikola et al., 2014 [121], reported that prolonged cold exposure for 5 to 8 hours was able to increase resting energy expenditure (REE) by 15%, and plasma glucose (30%) and FFA (70%) contributed to the observed increase in REE. Glucose disposal was increased in BAT/beige and whole-body glucose disposal was significantly increased. In diabetes mellitus type 2 subjects, 10 days of cold acclimation increased peripheral insulin sensitivity by 43%. Basal skeletal muscle glucose transporter type 4 (GLUT4) translocation was markedly increased and glucose uptake in skeletal muscle was increased after cold acclimation. It seems that cold exposure was able to increase energy expenditure and have beneficial effects on glucose metabolism supporting its role in treating obesity and related metabolic disorders in humans probably through an effect on beige activation.

Adipose derived stem cells can be induced to differentiate to beige adipocytes in many ways; the large amount of research in this area suggests a great number of potential drugs in the next few years. Objectively, beige adipocytes in the adult have been shown having a beneficial effect on both the sensitivity to insulin and the reduction in body weight [122]. Although the mechanisms by which these effects occur have not been sufficiently elucidated, using the modulation of the beige adipocyte in adipose cell progenitors in the adult is clearly a therapeutic approach, especially for type 2 diabetes mellitus. Taking into account that diabetes mellitus type 2 has two key pathophysiological components, the reduction of insulin secretion and the peripheral resistance to insulin action, a change in the sensitivity to insulin mediated by an increase in the number of beige adipocytes can be a great therapeutic strategy. Additionally, a reduction in body weight may lead to a reduced load on the activity of the pancreas. Studies in mouse models have demonstrated that the manipulation of the beige adipocytes is sufficient to alter energy expenditure and homeostasis.

Some experiments have shown that a reduction in glucose levels along with an increase in insulin sensitivity can be obtained with the induction of beige/brown fat in humans [121].

Certain therapeutic effects must be evaluated before the possible clinical use of beige adipocyte induction. First, it should be determined whether the metabolic effects of the beige adipocyte are subject to an increase in UCP1 or there are alternate metabolic pathways that could improve the condition of the adipose cells [123]. It is possible that the stimulated beige adipocytes, like the brown adipocytes, not only are heat generators but also contribute to the improved metabolism of glucose and lipids through the secretion of specific factors. Another necessary aspect addresses whether there is a more specific detector for the presence of beige adipocytes in the organism; although F-FDG-PET has been improved considerably, it is desirable to develop new tools or instruments that can quantify the amount of beige adipocyte tissue in the body.

The biology of beige adipocytes is so novel that it is necessary to gain an understanding of the physiological conditions, the number of days that the cells can survive, the elements that may be necessary to maintain the functionality of this cell type, and so on.

Finally, it is important to determine the specific factors that provide plasticity to beige adipose tissue, which makes possible their differentiation from a mature white adipose cell. The process of beiging from both the precursor adipose cells as well as from the white adipose cells may be a desirable element for weight reduction.

## Figures and Tables

**Table 1:** Characteristic of different kind of adipocyte tissue.

White adipocytes	Brown adipocyte	Beige adipocyte ^1^Cold, TZD, FGF21, IL-4, IL-6
Unilocular adipocyte	Multilocular adipocytes	Unilocular → multilocular
Lipid storage (+++)	Lipid storage (+)	Lipid storage (+++) → Lipid storage (++)
Mitochondria (+)	Mitochondria (+++)	Mitochondria (+) → mitochondria (++)
Fatty acid oxidation (+)	Fatty acid oxidation (+++)	Fatty acid oxidation (+) → fatty acid oxidation (+++)
Respiratory chain (+)	Respiratory chain (+++)	Respiratory chain (+) → respiratory chain (+++)
UCP1 (−)	UCP1 (+++)	UCP1 (−) → UCP1 (+++)
PGC-1*α* (+)	PGC-1*α* (+++)	PGC-1*α* (+) → PGC-1*α* (+++)
Markers: resistin, ASC-1, FAB4	Markers: Zic1, Lhx8, Eva1, Pdk4	Markers: CD137, Tbx1, Cited1, Tmem26, CIDEA

^1^Conditions that stimulate the thermogenic activity of beige adipocytes.

TZD: thiazolidinedione; FGF21: fibroblast growth factor 21; UCP1: uncoupling protein 1; PGC-1*α*: peroxisome proliferator-activated receptor-gamma coactivator alpha 1; ASC-1: adipocyte-specific cell surface protein; FABP4: fatty acid binding protein 4; Zic1: zinc finger protein 1; Lhx8: LIM/homeobox protein; Eva1: epithelial V-like antigen 1; Pdk4: pyruvate dehydrogenase kinase 4; CD 137: cluster of differentiation 137; Tbx1: T-box transcription factor 1; Cited1: Cbp/P300-interacting transactivator 1; Tmem26: transmembrane protein 26; CIDEA: Cell death-inducing DFFA-like effector.
